# Calibrating the Performance of SNP Arrays for Whole-Genome Association Studies

**DOI:** 10.1371/journal.pgen.1000109

**Published:** 2008-06-27

**Authors:** Ke Hao, Eric E. Schadt, John D. Storey

**Affiliations:** Rosetta Inpharmatics, Seattle, Washington, United States of America; University of Michigan, United States of America

## Abstract

To facilitate whole-genome association studies (WGAS), several high-density SNP genotyping arrays have been developed. Genetic coverage and statistical power are the primary benchmark metrics in evaluating the performance of SNP arrays. Ideally, such evaluations would be done on a SNP set and a cohort of individuals that are both independently sampled from the original SNPs and individuals used in developing the arrays. Without utilization of an independent test set, previous estimates of genetic coverage and statistical power may be subject to an overfitting bias. Additionally, the SNP arrays' statistical power in WGAS has not been systematically assessed on real traits. One robust setting for doing so is to evaluate statistical power on thousands of traits measured from a single set of individuals. In this study, 359 newly sampled Americans of European descent were genotyped using both Affymetrix 500K (Affx500K) and Illumina 650Y (Ilmn650K) SNP arrays. From these data, we were able to obtain estimates of genetic coverage, which are robust to overfitting, by constructing an independent test set from among these genotypes and individuals. Furthermore, we collected liver tissue RNA from the participants and profiled these samples on a comprehensive gene expression microarray. The RNA levels were used as a large-scale set of quantitative traits to calibrate the relative statistical power of the commercial arrays. Our genetic coverage estimates are lower than previous reports, providing evidence that previous estimates may be inflated due to overfitting. The Ilmn650K platform showed reasonable power (50% or greater) to detect SNPs associated with quantitative traits when the signal-to-noise ratio (SNR) is greater than or equal to 0.5 and the causal SNP's minor allele frequency (MAF) is greater than or equal to 20% (N = 359). In testing each of the more than 40,000 gene expression traits for association to each of the SNPs on the Ilmn650K and Affx500K arrays, we found that the Ilmn650K yielded 15% times more discoveries than the Affx500K at the same false discovery rate (FDR) level.

## Author Summary

Advances in SNP genotyping array technologies have made whole-genome association studies (WGAS) a readily available approach. Genetic coverage and the statistical power are two key properties to evaluate on the arrays. In this study, 359 newly sampled individuals were genotyped using Affymetrix 500K and Illumina 650Y SNP arrays. From these data, we obtained new estimates of genetic coverage by constructing a test set from among these genotypes and individuals that is independent from the SNPs and individuals used to construct the arrays. These estimates are notably smaller than previous ones, which we argue is due to an overfitting bias in previous studies. We also collected liver tissue RNA from the participants and profiled these samples on a comprehensive gene expression microarray. The RNA levels were used as a large-scale set of quantitative traits to calibrate the relative statistical power of the commercial arrays. Through this dataset and simulations, we find that the SNP arrays provide adequate power to detect quantitative trait loci when the causal SNP's minor allele frequency is greater than 20%, but low power is less than 10%. Importantly, we provide evidence that sample size has a greater impact on the power of WGAS than SNP density or genetic coverage.

## Introduction

It has been estimated that the human genome contains more than 5 million common single nucleotide polymorphisms (SNPs) with minor allele frequencies (MAF) ≥10% [1]–[3], and 7.5 million common SNPs with MAF ≥5% [4]. These SNPs may account for the genetic risk of many common human disorders. Recently, high-density SNP arrays have been introduced to allow researchers to conduct whole-genome association studies (WGAS). These SNP array platforms are often benchmarked by their genetic coverage and statistical power [4],[5]. Here, genetic coverage of an array platform is defined as the fraction of common SNPs (MAF≥5%) exceeding a predefined correlation threshold with at least one SNP typed by the array. Statistical power in this setting measures the likelihood to detect a statistically significant association between a truly associated SNP marker and a trait.

There are two strategies to building whole-genome SNP arrays. One is to randomly select SNPs that are relatively evenly spaced across the genome, not taking into account the inter-SNP linkage disequilibrium (LD) patterns, such as the Affymetrix 100K and 500K SNP arrays (denoted as Affx100K and Affx500K in this article, respectively) [4],[5]. The other is to select “tag SNPs” based on measures of LD chosen to maximize genetic coverage, such as Illumina HumanHap-300 -550K and -650Y arrays (denoted as Ilmn300K, Ilmn550K and Ilmn650K, respectively) [4],[5]. These tag SNP microarrays were developed using the public HapMap dataset [2],[3],[6].

The identification of tag SNPs is essentially a feature selection problem. It has been well established in the machine learning field that using an independently sampled test dataset is necessary to guarantee an unbiased assessment of the selected features' operating characteristics. It has also been shown that if the evaluation takes place on the training dataset itself, then the quality of the features' performance is often anti-conservatively over-estimated, commonly referred to as the overfitting problem [7],[8]. This problem exists in the context of identifying tag SNPs in two ways: (i) SNP overfitting, where the same set of SNPs are employed in both the training and evaluation steps; and (ii) sample overfitting, where the same set of subjects are used in both the training and evaluation steps.

The key operating characteristics of several whole-genome SNP arrays have been evaluated recently on HapMap data [4],[5],[9]. These studies may have been susceptible to both types of overfitting because the HapMap subjects were used to select tag SNPs; these same subjects and tag SNPs were then used in estimating the genetic coverage. Additionally, the small sample size of the HapMap data may limit the accuracy of estimates of statistical power, an operating characteristic that is critical for WGAS. Here, we present a study with the following characteristics to overcome these potential limitations: (i) the study subjects have been newly and independently sampled, and thus represent an independent sample from HapMap individuals, (ii) we have available a new set of genotyped SNPs which were sampled independently from HapMap data, and (iii) the sample size is relatively larger.

We utilized two commercially available high-density SNP arrays on an American Caucasian cohort to obtain estimates of genetic coverage for these different SNP panels that are robust to overfitting. The estimates we obtain in this cohort are lower than previous reports. In addition, liver RNAs were extracted and profiled on a comprehensive gene expression microarray. By simultaneously utilizing these thousands of gene expression traits scored on a fixed set of genetic backgrounds, we obtain estimates of the relative power of the different SNP genotyping arrays to detect quantitative trait SNPs of varying effect sizes [10]. We also directly quantify the impact of genetic coverage, SNP tagging, and sample size on the power of WGAS.

## Materials and Methods

### Liver Study Dataset

Human liver tissue samples were collected as described in a companion article [11]. In total, 359 American Caucasian subjects with known gender (heretofore called the “Liver Study subjects”) were successfully SNP genotyped and mRNA profiled.

#### Genotyping

DNA specimens were extracted and sent to Perlegen Sciences Inc. and Illumina Inc. for genotyping services using Affx500K and Ilmn650K, respectively. There was an overlap of >80,000 SNPs tiled on both SNP arrays. We found the two platforms gave highly consistent (98.7%) genotypes on their shared SNPs, suggesting both genotyping arrays are fairly accurate. We filtered out SNPs with MAF <5%, call rate <90%, or Hardy-Weinberg Test p-value <10^−4^. In total, 286K and 545K genotyped autosomal SNPs on Affx500K and Ilmn650K, respectively, were used in the analysis. Genotypes from Ilmn300K and Ilmn550K were also derived as subsets of the Ilmn650 data, resulting in 296K and 514K genotyped SNPs, respectively.

#### RNA expression profiling

Additionally, we purified RNA from the tissue samples and measured the 39,280 gene transcription levels using the Agilent platform. We adjusted the expression values for gender, age, and medical center by using a standard linear model. See Text S1 for specific details on expression profiling and preprocessing.

### HapMap Dataset

The HapMap data are comprised of 270 individuals from four ethnic groups: (i) 30 trios from the Yoruba group in Ibadan, Nigeria (YRI); (ii) 30 trios from the CEPH collection, which are Utah residents with Northern and Western Europe ancestry (CEU); (iii) 45 unrelated Han Chinese individuals from Beijing, China (CHB); and (iv) 45 unrelated individuals from Tokyo, Japan (JPT). The CHB and JPT samples are often considered as a single East Asian sample [3]. The HapMap project has genotyped more than 4 million SNPs, among which ∼2.2 million SNPs are common in CEU (MAF ≥5%) [4],[9]. Additionally, Affymetrix Inc. has genotyped these 270 individuals using Affx500K, and made these results publicly available.

### Identification of SNPs Independent from HapMap Data

Recall that the Affx500K platform harbors 90K common SNPs that were not utilized in the HapMap project (referred to here as Affx NonHapMap SNPs). The genotypes from the Affx500K platform measured on the 359 Liver Study subjects therefore provide two key sources of independent data: (i) genotypes of SNPs identified independently from the HapMap project (the Affx NonHapMap SNPs) and (ii) individuals sampled independently from the HapMap project. This allowed us to study SNP selection overfitting and sample overfitting, respectively, in calculating genetic coverage.

Given that SNPs on the Affx500K were randomly chosen, the 90K Affx NonHapMap SNPs can be considered as a random sample of the entire 5.3M NonHapMap SNPs. We therefore utilized these Affx NonHapMap SNPs as a set of SNPs identified independently from the HapMap data. Also note that the complementary Affx HapMap SNPs (SNPs on Affx500K that are included in HapMap data) represent a random subsample of the 2.2M HapMap SNPs. We utilized these two classes of SNPs genotyped in our independently sampled human cohort scored for thousands of gene expression traits to estimate the true genetic coverage of the different SNP panels as well as assess the power these panels afford to detect associations between SNPs and traits (details below).

### Estimating Genetic Coverage

Genetic coverage was calculated as the fraction of SNPs exceeding a pre-defined r^2^ cutoff (r^2^_cutoff_) with at least one SNP typed by the microarray [5]. Herein, we employed two widely used values of r^2^_cutoff_, 0.8 and 0.9. Results were qualitatively equivalent in the 0.7 to 0.9 range for r^2^_cutoff_.

### Assessing Overfitting of Previous Genetic Coverage Estimates

Overfitting of genetic coverage estimates was assessed separately for SNP overfitting and sample overfitting.

#### SNP overfitting

To systematically evaluate whether there has been SNP overfitting in estimated genetic coverage, we compared genetic coverage rates of Affx NonHapMap SNPs to Affx HapMap SNPs on the Illm650K platform. A higher genetic coverage of Affx HapMap SNPs is evidence for SNP overfitting. The SNPs represented on Ilmn300K and Ilmn550K are subsets of Ilmn650K, and we computed their genetic coverage in a similar manner.

#### Sample overfitting

To determine whether there has been sample overfitting in previous estimates, we compared genetic coverage of the CEU individuals versus the Liver Study individuals. A higher level of genetic coverage in the former is evidence of overfitting. We made this comparison among Affx HapMap SNPs, among Affx HapMap SNPs, and among all Affx SNPs combined. Evidence for sample overfitting was present for all three sets of SNPs. Finally, for both types of overfitting, we stratified the AffxSNPs into MAF bins, and computed coverage for each bin to investigate whether the overfitting bias is also a function of MAF.

### Statistical Power for Mapping Continuous Traits

The statistical power of the SNP array platforms for WGAS were first estimated from simulation studies. First, we randomly selected a SNP from Affx500K and used its genotype to simulate trait value. Over the range of simulations, SNP genotypes from both Affx HapMap SNPs and Affx NonHapMap SNPs were utilized. We assumed the trait followed a Normal distribution N(μ,σ^2^), where σ^2^ was constantly set to 1 and μ varied among genotypes. We set (μ_AA_, μ_Aa_, μ_aa_) = (−0.5, 0, 0.5), (−0.25, 0, 0.25), or (−1, 0, 1) to investigate a range of signal strengths. Second, we conducted single-marker tests, which examined association between each SNP and each simulated trait. We surveyed three choices of α level (10^−5^, 10^−6^ and 10^−7^) that are reasonable for WGAS. Kruskal-Wallis and Spearman rank correlation tests were employed because these non-parametric methods were robust to the underlying genetic model and trait distribution, thereby allowing our simulation to be useful for non-normal traits and non-additive models. We defined a “true discovery” to be any association detected within 200 kb of the causal SNP. We calculated statistical power (defined as the probability of calling any SNP within 200kb of the causal SNP significant) and the average number of true discoveries (NTD) over the set of simulated datasets. Two million simulation runs were conducted for each parameter setting.

### Statistical Power for Mapping Binary Traits

Again, we firstly simulated a binary phenotype using Affx HapMap SNPs and Affx NonHapMap SNPs, respectively. The genetic model was specified as disease prevalence = 25% and relative risk = 3. Once the phenotypes were generated, we randomly picked 75 cases and 75 controls from the 359 subjects to construct a balanced case-control study. These simulation parameters were chosen to ensure statistical power in a range easy to compare. Since the sample size was relatively small, Fisher's exact test was applied. Two million simulation loops were run for each scenario.

### Association Mapping of Expression Traits

Using the same procedure as above, single-marker association tests were conducted to detect the expression quantitative trait loci (eQTL) for each of the ∼40,000 gene expression traits measured. Furthermore, we repeated the tests on three permuted gene expression datasets and calculated the false discovery rate (FDR). In each permutation run, we first randomized the patient IDs in the expression file, breaking any association between expression traits and genotypes while leaving the respective correlation structures among gene expression traits and SNP genotypes intact. Second, we repeated the association tests for every expression trait and genotype pair, resulting a set of null statistics for each permutation. A standard FDR estimator was then applied to the resulting association statistics, as previously carried out on observed and permutation null statistics [12]. Because the entire set of null statistics were used to calculate the q-value for each test, we were able to use only three permutations and still retain stable significance results.

## Results

### Genetic Coverage

**Figure 1:**
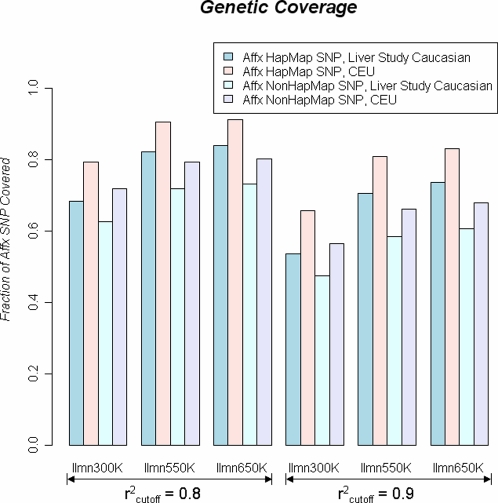
Plot of genetic coverage of Affx HapMap SNPs and Affx NonHapMap SNPs were calculated among HapMap CEU subjects and liver study subjects, respectively. The effect of SNP overfitting and sample overfitting can be seen.

Based on the Liver Study subjects, we obtained new estimates of genetic coverage for the Illumina and Affymetrix SNP genotyping arrays (Materials and Methods), which are robust to overfitting. Evidence of SNP overfitting in previous estimates can be seen in Figure 1 by comparing the genetic coverage of Affx HapMap SNPs to that of Affx NonHapMap SNPs based on the genotypes from the Ilmn650K array. (Materials and Methods). As can be seen, SNP overfitting is present in genetic coverage estimates derived from both HapMap CEU individuals and Liver Study individuals. Interestingly, the magnitude of SNP overfitting was similar when repeating the analysis on the Ilmn550K and Ilmn300K arrays (Table S1).

By comparing estimates of genetic coverage derived from HapMap CEU to those from Liver Study subjects (Materials and Methods), we also found evidence for the existence of sample overfitting (Figure 1 and Table S1). For example, the Ilmn300K platform's genetic coverage was reported to be 9% higher in CEU individuals than when we make the sample calculation on the Liver Study subjects. In contrast, the magnitude of sample overfitting was smaller in the Ilmn550K and Ilmn650K sets. This phenomenon could be explained by the degree of redundancy in the tag SNP sets. The first genome-wide tag SNP array, Ilmn300K, harbors a “lean” set of 317K tag SNPs optimized only in CEU. As a drawback, these 317K SNPs contained less redundancy and exhibited less transferability. The Ilmn550K and Ilmn650K were developed on multiple ethnic groups [9], and their tag SNP sets had higher degree of redundancy, resulting in better transferability.

By comparing genetic coverage of the Affx HapMap SNPs in CEU to Affx NonHapMap SNPs in Liver Study subjects, we measured the combined size of the two types of overfitting to be ∼18%. Furthermore, we formed weighted estimates by taking the weighted average of the coverage on HapMap SNPs (*w* = 2.2/7.5) and that on NonHapMap SNPs (*w* = 5.3/7.5). Among the Liver Study Caucasian subjects, the Ilmn300K and Ilmn650K's weighted genetic coverage was 64% and 76% at r^2^_cutoff_ = 0.8, which is lower than previous reports (79% and 90%, respectively; http://www.cidr.jhmi.edu/human_gwa.html). Furthermore, we found that the tagSNP arrays cover low MAF SNPs (e.g. MAF<15%) worse than the high MAF ones (e.g., MAF≥15%), and importantly, the overfitting bias appears to be more severe for the low MAF range (Figure S1 and Table S1).

### Statistical Power

A WGAS requires genotyping thousands of subjects, which is expensive at current genotyping costs [13],[14]. To conserve resources, many WGAS are adopting a two-stage design in which a small sample of subjects (e.g., a few hundred) are genotyped on all markers in stage 1, and a proportion of these markers are genotyped on a much larger sample in stage 2 [13],[14]. Studies have shown that this strategy may preserve much of the power of the corresponding one-stage design and minimizes the genotyping cost [14]. In our study, N = 359 is a reasonable sample size for the stage 1 WGAS [14]–[16]. The SNP arrays showed reasonable power to detect SNPs associated with quantitative traits when the SNR >0.5, but limited power when SNR ≤0.25 (Table S2).

**Figure 2:**
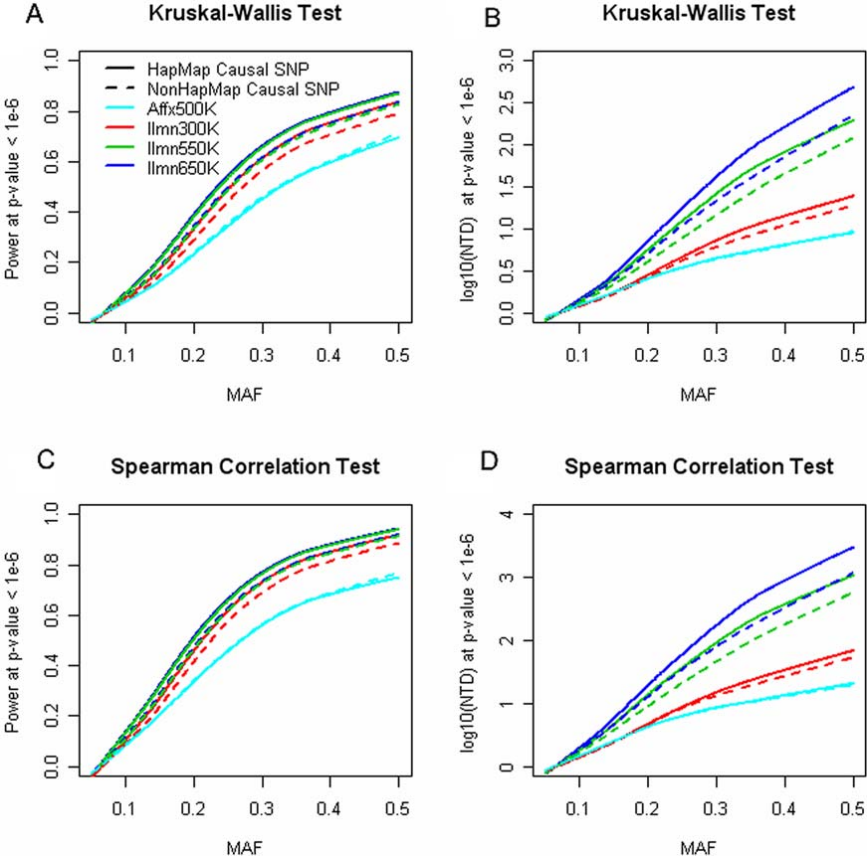
On the simulated trait values, the statistical power and NTD (number of true discoveries) were estimated for the Affymetrix 500K and Illumina tag SNP arrays.

In simulating SNPs causal for a quantitative trait, we assumed μ_AA_<μ_Aa_<μ_aa_, so that the Spearman rank correlation test gave higher statistical power and larger NTD than the Kruskal-Wallis rank-sum test. In a separate set of simulations (Figure S2), we found the Kruskal-Wallis test was conservative in the small p-value range (e.g. p-value<0.05). Focusing on SNR = 0.5, we found that the statistical power was highly related to the causal SNP's MAF (Figure 2). For example, when MAF≥20% the Ilmn650K had over 50% power to detect associations with quantitative traits (Spearman rank correlation test). When the MAF ≤10%, little power could be achieved (Figure 2).

**Figure 3:**
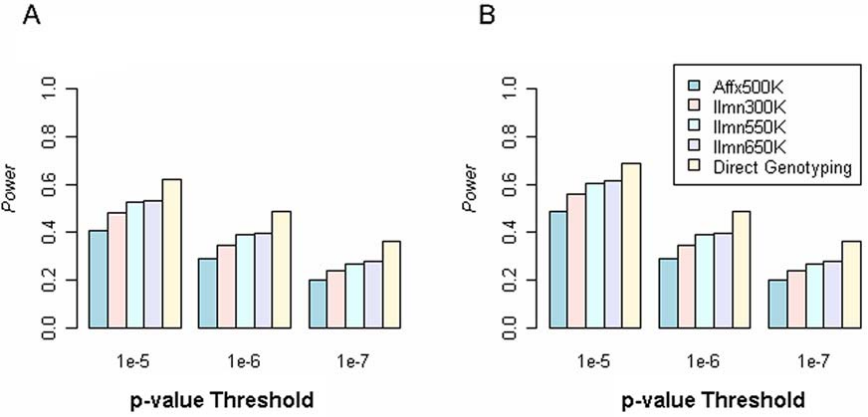
Weighted estimates for statistical power by taking the weighted average of the power on HapMap causal SNPs (weight = 2.2/7.5) and that on NonHapMap causal SNPs (weight = 5.3/7.5). (A) Kruskal-Wallis tests. (B) Spearman rank correlation tests. Further, we quantified the power of “direct genotyping,” where association tests were conducted on causal SNPs. This represents an upper bound on statistical power in WGAS.

Not surprisingly, we observed the Affx500K to exhibit less power than the Ilmn650K, which could be explained the fact that the Affx500K platform contains fewer SNPs and/or that the Ilmn650K platform has higher genetic coverage. Because the Ilmn550K and Ilmn650K platforms had similar genetic coverage in Caucasians, they showed nearly the same statistical power (Figures 2 and 3). In contrast, the Ilmn650K platform offered a larger number of true discoveries (NTD), indicating more significant SNPs were detected around the true causal SNP.

Interestingly, the power of Illumina arrays differed when identifying associations with quantitative traits simulated using Affx HapMap SNPs and Affx NonHapMap SNPs which was essentially the overfitting effect. For example, using Kruskal-Wallis test and a p-value threshold of α = 10^−6^, Ilmn550K showed 37% power in detecting NonHapMap causal SNPs and 43% power in detecting HapMap causal SNPs (Table S2B), which translated into a difference of 6% in power, likely due to an overfitting bias. We also surveyed other genetic models as well as significance thresholds, and observed considerable SNP overfitting effects (Table S2A, S2C, and S2D).

In a WGAS, a large number of hypothesis tests are conducted, so that statistical significance measures such as the FDR need to be carefully assessed. In our simulation, the true causal SNPs were known. When significant associations were detected >1Mb away from the causal SNP or on a different chromosome, we regarded them as false discoveries. The number of false discoveries (NFD) was proportional to the number of SNPs employed in the WGAS, with NFD_Affx500K_<NFD_Ilmn300K_<NFD_Ilmn550K_<NFD_Ilmn650K_. Comparing Table S2B and Table S3, we found the FDR was in a manageable range. For example, at a *P*<10^−5^, Ilmn550K gave an average of 2.51 NFD and 1.69 NTD using the Kruskal-Wallis test. At a *P*<10^−6^, 0.20 NFD and 1.06 NTD were observed; and at a more stringent *P*<10^−7^, the FDR was even smaller, suggesting 10^−6^ or 10^−7^ as an appropriate significant cutoff in WGAS or initial screening (Figure S3).

Finally, we conducted association tests on actual traits, namely RNA expression levels or “expression traits.” It has been shown that comparing the ND from many related traits, all conditional on the same set of individuals (i.e., genetic backgrounds), can lead to meaningful empirical power comparisons, where simple models often used for simulations do not have to be assumed [10]. Specifically, by considering the relative ND among different approaches at the same error rate, we are able to estimate the relative levels of power, without having to specifically identify which among the discoveries are true discoveries [17].

**Figure 4:**
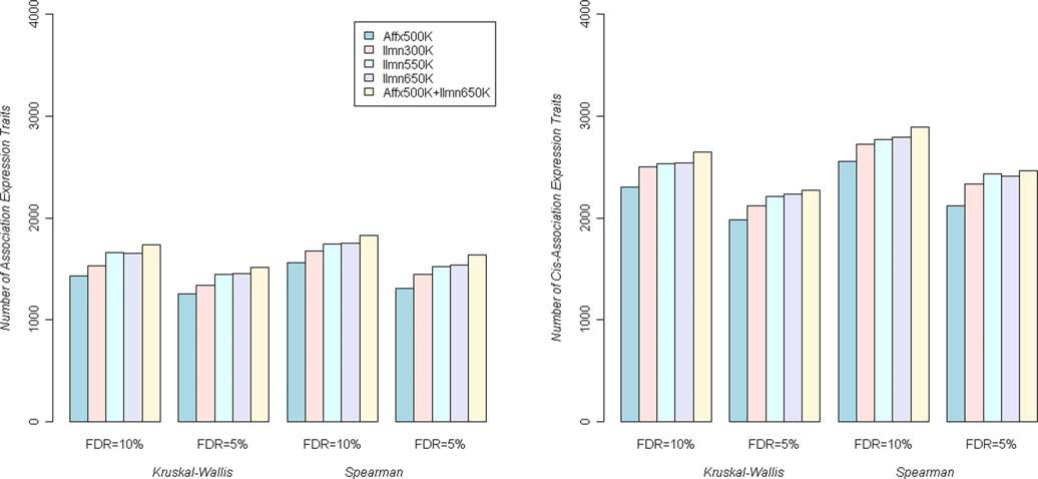
Tests of association on liver gene expression traits. (A) Number of gene expression traits that were associated with SNPs on Affymetrix and Illumina microarrays at fixed FDR levels. (B) We restricted the association tests to SNPs within 1 Mb range of the gene and present the number of *cis*-associating gene expression traits at a given FDR level.

The number of discoveries (ND) obtained on both observed data and permuted data followed the same pattern: Ilmn650K>Ilmn550K>Ilmn300K>Affx500K. Because the true and false discoveries were no longer distinguishable, we could not directly infer the SNP arrays' statistical power using ND. Instead, we compared the relative power using ND conditioning on FDR (Figures 4 and S3). The Ilmn650K slightly outperformed the Ilmn550K, indicating the “100K YRI SNPs” on Ilmn650K [9] benefited Caucasian studies although they were selected on HapMap YRI. Compared to Affx500K, Ilmn650K discovered 15% more genes that were associated with at least one SNP (FDR = 10%).

After filtering SNPs based on MAF, call rate, and HWE p-values (Materials and Methods), a similar number of SNPs on Affx500K and Ilmn300K (286K and 296K SNPs, respectively) were included in the analysis, which provided an opportunity for a head-to-head comparison between random SNPs and tag SNPs on these expression traits. Unexpectedly, the Affx500K outperformed Ilmn300K in term of ND (Figure S4, upper panels), indicating random SNPs detected more significant associations than the same number of tag SNPs at the same FDR levels. However, the Ilmn300K captured more quantitative traits (Figures 4 and S4, lower panels). One explanation could be that the Affx500K SNPs, clustered on Nsp and Sty restriction fragments rather than strategically spread on human genome, tended to capture certain signals repeatedly but missed other associations.

As a novel feature of our study, we were also able to investigate the power of combining Affx500K and Ilmn650K arrays for a single analysis (Figure 4). Since the location of each expressions trait is known, this allows us to focus on the *cis*-associations. In brief, for a given expression trait, only SNPs within ±1 Mb of the corresponding gene are tested. By taking these steps, the number of tests is substantially reduced and the statistical power increased, illustrated in more *cis*-association discoveries in Figure 4 right panel comparing to the left panel. The numbers of *cis*-association genes also reflect the relative power. Consistent with Figure S4, the Illumina tag SNP arrays are more powerful than Affx500K. For example, using Affx500K as the reference, 650K panels' relative power is 110%, in detecting *cis*-association. Surprisingly, Affx500K+Ilmn650K (relative power = 115%) only slightly outperforms Ilmn650K, indicating the limited return of adding additional SNPs on top of Ilmn650K.

### Sample Size Versus Genetic Coverage

We collected 68 additional liver samples from Caucasian donors. We performed RNA expression profiling as before and obtained SNP genotypes using the Affy500K array only. We then we pooled the sample (re-normalizing for gender, age, and medical center, and batch) and reran the association tests on the Affx500K SNPs. Surprisingly, this increase in sample size (19%) results in 31% and 33% more *cis*-association discoveries (at 5% and 10% FDR, respectively), implying a respective 31% and 33% boost in relative power. In contrast, conditioning on the same sample size, Ilmn650K yields about 10% more *cis*-associations than Affy500K. This is potentially an important observation that sample size has a more profound impact on statistical power than the difference in genetic coverage among current SNP arrays. Since arrays vary greatly in price, and argument has been raised whether to choose high genetic coverage arrays or cheaper ones and run more subjects.

## Discussion

Whole-genome association studies using high-density SNP arrays are viewed as a powerful approach to elucidating the genetic bases of common human diseases. We provided a novel investigation of two key properties for determining the performance of SNP array genotyping platforms in WGAS: genetic coverage and statistical power. The availability of (i) 90K genotyped SNPs which were identified independently from the HapMap, (ii) a new, independently sampled cohort of subjects, and (iii) thousands of related “expression traits” measured simultaneously on these subjects, yielded the opportunity to provide new insights into genetic coverage and statistical power, and make comparisons to previous results.

Two strategies are usually taken in selecting SNPs and constructing genome wide arrays: random SNPs and tag SNPs. These strategies might result in different levels of performance in terms of genetic coverage and statistical power [4]. Regardless of the selection algorithms used, the performance of tag SNPs is most accurately assessed by using a validation dataset independent of the training set [18]. In this article, we systematically investigated two sources of overfitting (SNP overfitting and sample overfitting, respectively) and derived new genetic coverage estimates robust to these two types of overfitting. As a caveat, Affymetrix developed Affx500K array by screening the dbSNP database, which tends to document frequent SNPs rather than rare SNPs. As the result, the Affx SNPs have higher MAF than the totality of SNPs in the human genome, and our weighted genetic coverage estimates may be somewhat upwardly biased. However, this bias is an issue distinct from bias due to overfitting.

Since there are a limited number of common SNPs in the human genome, tag SNPs selected from the complete set (e.g., the 7.5 million common SNPs) would be robust to SNP overfitting in assessing genetic coverage. At the current stage, tag SNPs are usually selected from an incomplete initial SNP set (i.e., HapMap SNPs), and the remaining SNPs (i.e., 5.3M NonHapMap SNPs) would be “hidden” from the training procedure. Previous simulation studies showed that 26% of the common ENCODE SNPs in CEU had no good proxies (*r^2^*≥0.8) among the “pseudo” HapMap I SNPs [19]. This implies that these 26% SNPs would have an extremely low likelihood of being captured by tag SNPs (e.g. Ilmn300K) selected using HapMap I. Using empirical datasets, researches studied the SNP overfitting problem in a diverse set of ethnic groups around the world [3],[5],[20],[21]. However, these studies faced the limitation of small chromosomal regions and they didn't consider overfitting in the context of statistical power.

Our study employed 359 individuals, which provided adequate levels statistical power for moderate genetic effects (e.g., SNR = 0.5). Certainly, larger sample size is necessary to detect weaker effects (e.g., SNR = 0.25). Illumina tag SNP arrays were designed to capture common HapMap SNPs. Therefore, most of these tag SNPs have MAF ≥5%. In contrast, Affx500K harbors over 100K rare SNPs (MAF ≤5%). Because we used common SNPs to simulate quantitative traits, the Affx500K rare SNPs provided little statistical power and were therefore excluded from the analysis. Rare SNPs might be useful when the disease-causing polymorphism was also rare. However, Figure 2 showed WGAS may have limited power in such scenarios.

Our SNP filtering resulted in similar number of SNPs on Affx500K and Ilmn300K, which enabled a fair comparison between random SNPs and tag SNPs. We found random SNPs actually achieved larger ND (Figure S4), but many of which were redundant. In another words, Affx500K tended to capture the same signal repeatedly. In contrast, more traits were in association with at least one SNP on Ilmn300K, indicating tag SNPs were more efficient in WGAS. Such a finding was consistent to our simulation study, shown Figures 2 and 3, where Ilmn300K outperformed Affx500K in terms of statistical power.

Affymetrix and Illumina recently released 900K and 1M SNP arrays, respectively. These new products will further enhance WGAS. In evaluating their performance, we recommend utilizing independent test sets as we have done here. Given that we did not utilize these new arrays, we were not able to calibrate the genetic coverage and statistical power for the million-SNP arrays. However, there are a number of reasons why a major performance leap may not be expected. First, it has already been reported that the gain in coverage achieved by increasing the number of tag SNPs follows a pattern of diminishing returns [4],[19]. Second, the current tag SNP selection is still limited to occur within the HapMap dataset. As shown in Figure 1, this strategy results in an approximately 12% genetic coverage loss when applying to NonHapMap SNPs. In the ENCODE regions, ∼10% of the common SNPs had no good proxies (*r^2^*≥0.8) among the simulated HapMap II datasets, and those SNPs could only be adequately captured by searching beyond the HapMap data. In another words, the HapMap-derived tag SNP struggles to reach the 90% genetic coverage level. Through simulations, we were able to directly test the causal SNP, allowing us to calibrate the upper bound of a SNP array's performance in WGAS. At SNR = 0.5 (Figure 3), “direct genotyping” provided a gain of 8% more power than Ilmn650K, indicating current arrays are already capable of extracting a substantial level of genetic information. “Direct genotyping” provided a greater increase in power at SNR = 1 (Figure S5), but no extra power at SNR = 0.25.

**Figure 5:**
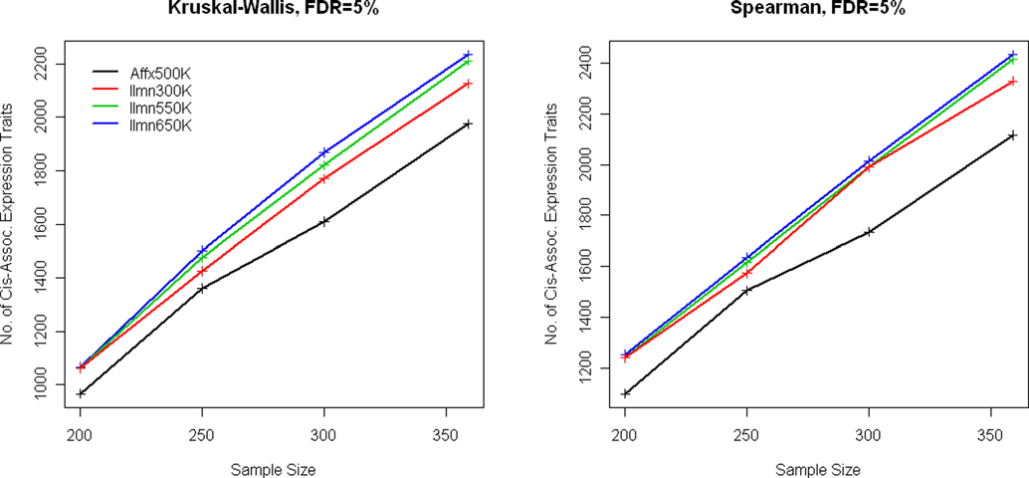
The number of significant *cis*-associations among the liver gene expression traits at FDR = % at varying sample sizes. The relative number of significant associations is an empirical estimate of the relative levels of power of the different platforms at different sample sizes.

It is important to continue to systematically quantify the trade-offs among genetic coverage, genotyping cost, and statistical power for WGAS. Based on our results, some conclusions are possible (Figure 5). For example, according to our results a study employing N = 300 subjects and the Affy500K platform offers higher power than a study employs N = 250 subjects and the Ilmn650K platform. This 20% increase in sample size (N = 300 vs. N = 250) provides more power than the 90% increase in the number of SNPs genotyped (286K SNPs vs. 545K SNPs). In scenarios where funding becomes the constraining factor, our results suggest that genotyping larger sample size with cheaper SNP arrays might achieve better statistical power. On the other hand, if the constraining factor is the number of subjects, then it appears that SNP arrays offering the largest genetic coverage should be employed.

## Supporting Information

Figure S1 We stratified Affx SNPs into MAF bins and computed the Illumina tag SNP array genetic coverage on HapMap CEU and Liver study subjects.
(28.44 MB TIF)

Figure S2 The null distribution of p-values for the Kruskal-Wallis test and Spearman Rank Correlation test. The p-value of Spearman test (p.spearman) follows the uniform distribution under the null, whereas the p-values of the Kruskal-Wallis test shows a lower density in the [0,0.05] range, indicating this test is conservative.
(0.33 MB TIF)

Figure S3 Using the expression traits, we surveyed a wide range of p-value cutoffs and the corresponding FDR values for simulated WGAS.
(1.23 MB TIF)

Figure S4 (A) and (B): the number of discovery (ND) observed in the expressiongenotype association screening. Please note that one expression trait sometimes showed significant association with multiple mutually proximal SNPs, because these SNPs were in strong LD. (C) and (D): the number of expression traits that showed at least one significant association with SNP(s).
(1.00 MB TIF)

Figure S5 The statistical power of the Affymetrix array, Illumina arrays, and “direct genotyping.” (A) SNR = 1/4 and Kruskal-Wallis test; (B) SNR = 1/4 and Spearman rank correlation test; (C) SNR = 1 and Kruskal-Wallis test; (D) SNR = 1 and Spearman rank correlation test.
(0.63 MB TIF)

Table S1 Genetic Coverage of tagSNP Arrays.
(0.11 MB DOC)

Table S2 Power and Number of Discoveries (ND) of Whole Genome SNP Genotyping Products.
(0.21 MB DOC)

Table S3 Number of False Discoveries in Mapping Quantitative Trait.
(0.04 MB DOC)

Text S1 Supplementary Materials.
(0.03 MB DOC)
