# Supplementary figures and images for: Calibrating the Performance of SNP Arrays for Whole-Genome Association Studies
Source: PLoS Genet. 2008 Jun 27;4(6):e1000109. doi: 10.1371/journal.pgen.1000109 (PMC2432039; doi:10.1371/journal.pgen.1000109)

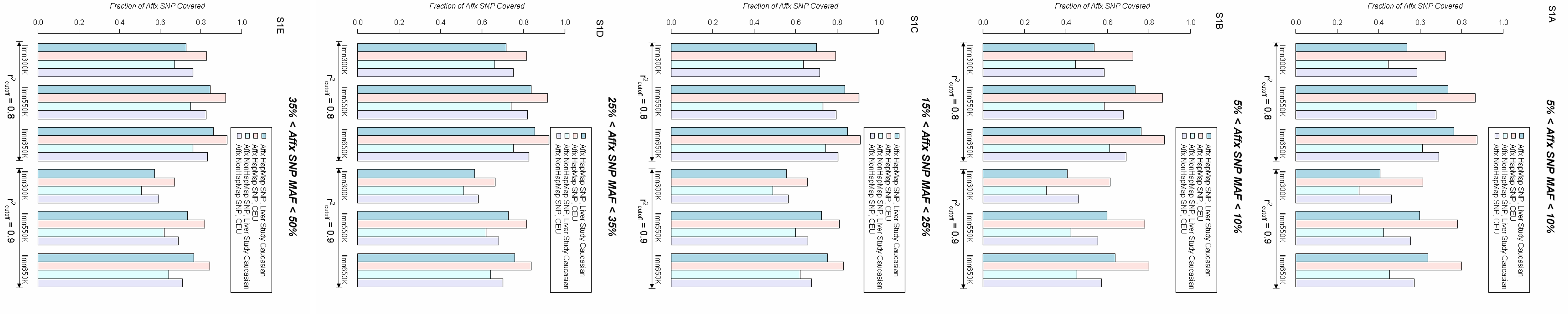

Supplement: Figure S1 — We stratified Affx SNPs into MAF bins and computed the Illumina tag SNP array genetic coverage on HapMap CEU and Liver study subjects. (28.44 MB TIF) [file pgen.1000109.s001.tif]

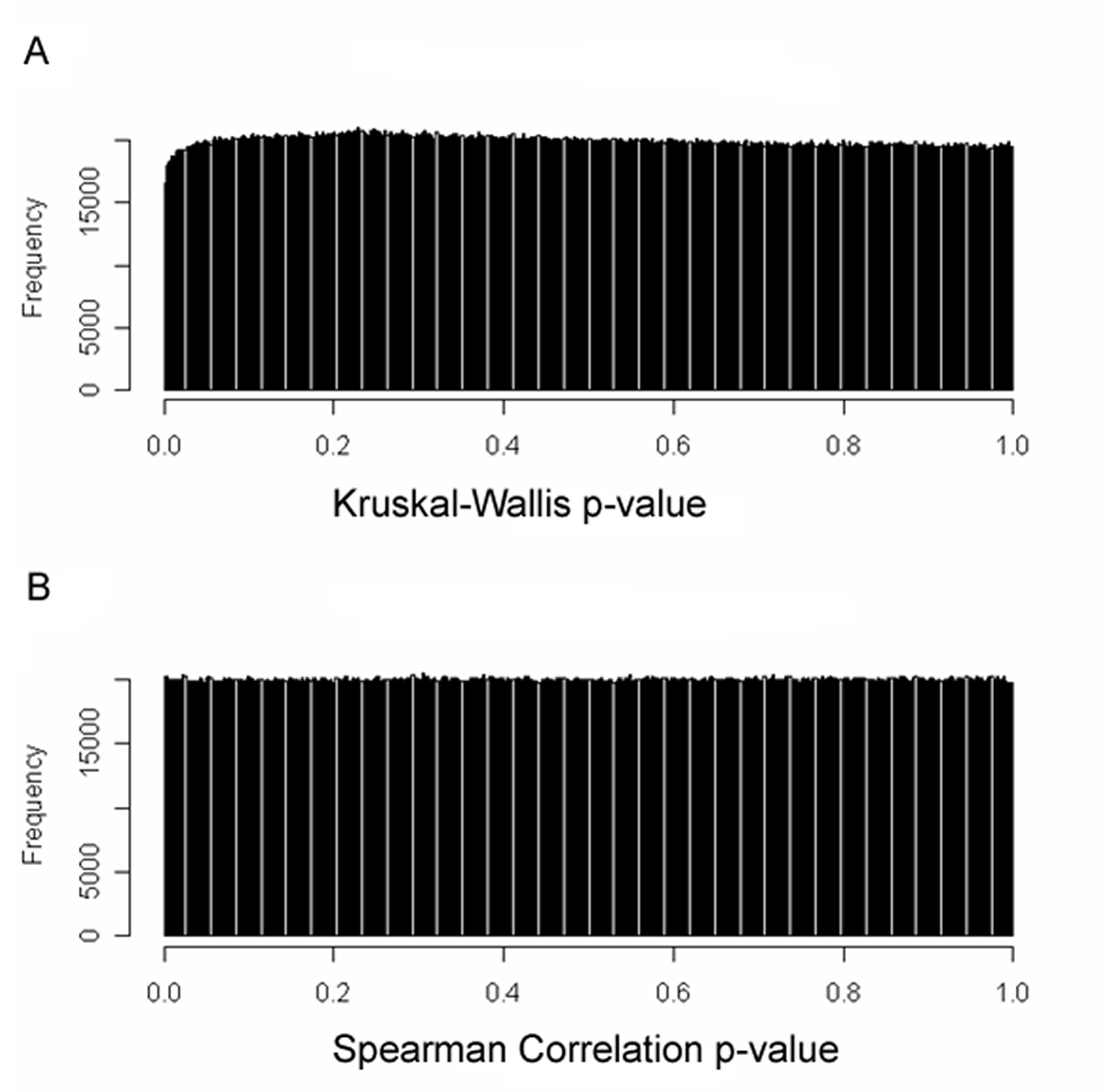

Supplement: Figure S2 — The null distribution of p-values for the Kruskal-Wallis test and Spearman Rank Correlation test. The p-value of Spearman test (p.spearman) follows the uniform distribution under the null, whereas the p-values of the Kruskal-Wallis test shows a lower density in the [0,0.05] range, indicating this test is conservative. (0.33 MB TIF) [file pgen.1000109.s002.tif]

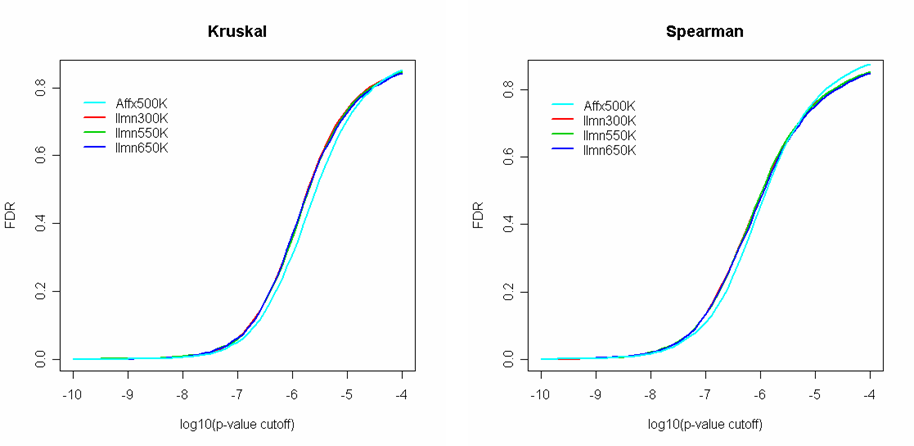

Supplement: Figure S3 — Using the expression traits, we surveyed a wide range of p-value cutoffs and the corresponding FDR values for simulated WGAS. (1.23 MB TIF) [file pgen.1000109.s003.tif]

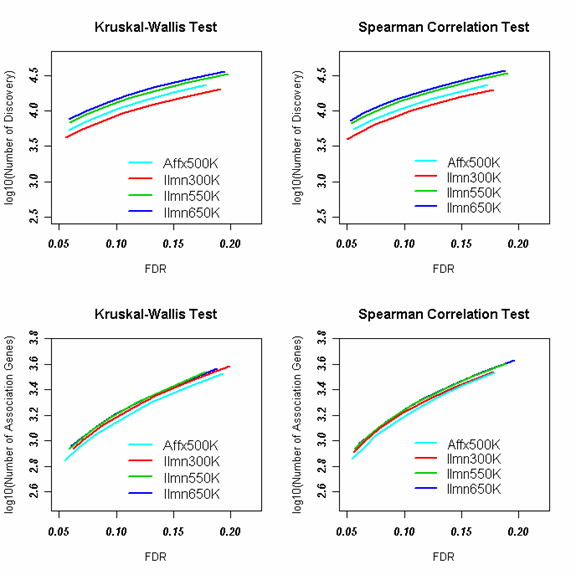

Supplement: Figure S4 — (A) and (B): the number of discovery (ND) observed in the expressiongenotype association screening. Please note that one expression trait sometimes showed significant association with multiple mutually proximal SNPs, because these SNPs were in strong LD. (C) and (D): the number of expression traits that showed at least one significant association with SNP(s). (1.00 MB TIF) [file pgen.1000109.s004.tif]

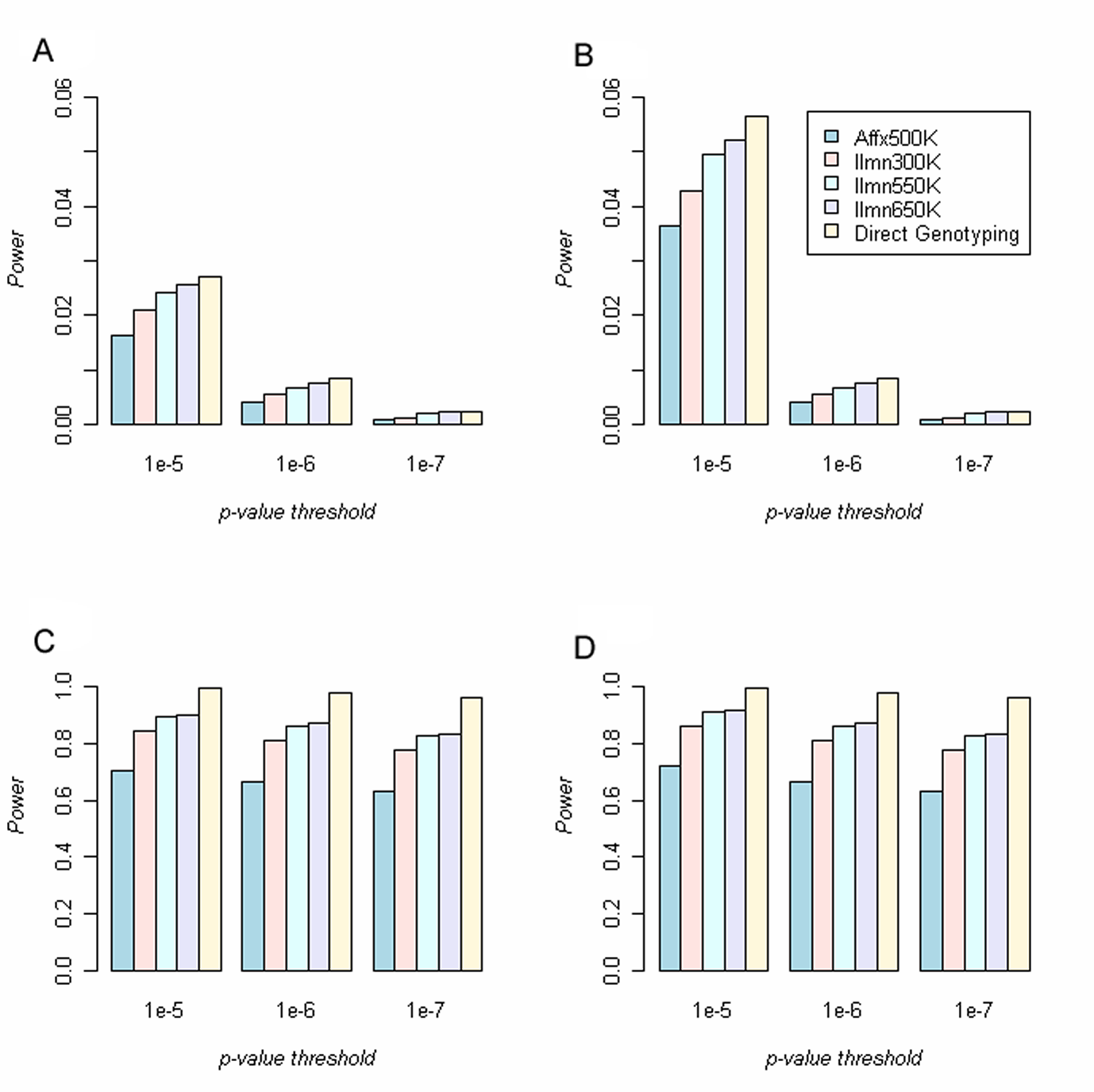

Supplement: Figure S5 — The statistical power of the Affymetrix array, Illumina arrays, and “direct genotyping.” (A) SNR = 1/4 and Kruskal-Wallis test; (B) SNR = 1/4 and Spearman rank correlation test; (C) SNR = 1 and Kruskal-Wallis test; (D) SNR = 1 and Spearman rank correlation test. (0.63 MB TIF) [file pgen.1000109.s005.tif]
