# Supplementary material for: Calibrating the Performance of SNP Arrays for Whole-Genome Association Studies
Source: PLoS Genet. 2008 Jun 27;4(6):e1000109. doi: 10.1371/journal.pgen.1000109 (PMC2432039; doi:10.1371/journal.pgen.1000109)
Supplement: Table S1 — Genetic Coverage of tagSNP Arrays. (0.11 MB DOC) [file pgen.1000109.s006.doc]

**Supplemental Table 1(a). Genetic Coverage of tagSNP Arrays on common AffxSNPs (MAF≥5%)**

|  | Affx HapMap SNP | | Affx NonHapMap SNP | |
| --- | --- | --- | --- | --- |
| r^2^_cutoff_ = 0.8 | Liver Study Caucasian | CEU | Liver Study Caucasian | CEU |
| Ilmn300K | 0.683 | 0.795 | 0.626 | 0.72 |
| Ilmn550K | 0.822 | 0.906 | 0.719 | 0.794 |
| Ilmn650K | 0.84 | 0.913 | 0.733 | 0.802 |
| r^2^_cutoff_ = 0.9 |  |  |  |  |
| Ilmn300K | 0.536 | 0.657 | 0.474 | 0.564 |
| Ilmn550K | 0.705 | 0.81 | 0.585 | 0.661 |
| Ilmn650K | 0.737 | 0.832 | 0.608 | 0.679 |

**Supplemental Table 1(b). Genetic Coverage of tagSNP Arrays on AffxSNPs that 10%>MAF≥5%**

|  | Affx HapMap SNP | | Affx NonHapMap SNP | |
| --- | --- | --- | --- | --- |
| r^2^_cutoff_ = 0.8 | Liver Study Caucasian | CEU | Liver Study Caucasian | CEU |
| Ilmn300K | 0.537 | 0.723 | 0.447 | 0.585 |
| Ilmn550K | 0.734 | 0.866 | 0.584 | 0.677 |
| Ilmn650K | 0.764 | 0.876 | 0.611 | 0.69 |
| r^2^_cutoff_ = 0.9 |  |  |  |  |
| Ilmn300K | 0.406 | 0.614 | 0.305 | 0.462 |
| Ilmn550K | 0.599 | 0.781 | 0.424 | 0.554 |
| Ilmn650K | 0.637 | 0.801 | 0.453 | 0.571 |

**Supplemental Table 1(c). Genetic Coverage of tagSNP Arrays on AffxSNPs that 15%>MAF≥10%**

|  | Affx HapMap SNP | | Affx NonHapMap SNP | |
| --- | --- | --- | --- | --- |
| r^2^_cutoff_ = 0.8 | Liver Study Caucasian | CEU | Liver Study Caucasian | CEU |
| Ilmn300K | 0.672 | 0.767 | 0.595 | 0.669 |
| Ilmn550K | 0.819 | 0.893 | 0.701 | 0.757 |
| Ilmn650K | 0.834 | 0.9 | 0.715 | 0.765 |
| r^2^_cutoff_ = 0.9 |  |  |  |  |
| Ilmn300K | 0.524 | 0.653 | 0.446 | 0.535 |
| Ilmn550K | 0.697 | 0.808 | 0.553 | 0.631 |
| Ilmn650K | 0.726 | 0.827 | 0.575 | 0.646 |

**Supplemental Table 1(d). Genetic Coverage of tagSNP Arrays on AffxSNPs that 25%>MAF≥15%**

|  | Affx HapMap SNP | | Affx NonHapMap SNP | |
| --- | --- | --- | --- | --- |
| r^2^_cutoff_ = 0.8 | Liver Study Caucasian | CEU | Liver Study Caucasian | CEU |
| Ilmn300K | 0.702 | 0.793 | 0.637 | 0.718 |
| Ilmn550K | 0.838 | 0.906 | 0.733 | 0.796 |
| Ilmn650K | 0.852 | 0.913 | 0.746 | 0.804 |
| r^2^_cutoff_ = 0.9 |  |  |  |  |
| Ilmn300K | 0.557 | 0.657 | 0.490 | 0.565 |
| Ilmn550K | 0.725 | 0.811 | 0.600 | 0.660 |
| Ilmn650K | 0.754 | 0.831 | 0.623 | 0.678 |

**Supplemental Table 1(e). Genetic Coverage of tagSNP Arrays on AffxSNPs that 35%>MAF≥25%**

|  | Affx HapMap SNP | | Affx NonHapMap SNP | |
| --- | --- | --- | --- | --- |
| r^2^_cutoff_ = 0.8 | Liver Study Caucasian | CEU | Liver Study Caucasian | CEU |
| Ilmn300K | 0.716 | 0.815 | 0.661 | 0.752 |
| Ilmn550K | 0.839 | 0.917 | 0.742 | 0.82 |
| Ilmn650K | 0.855 | 0.924 | 0.753 | 0.827 |
| r^2^_cutoff_ = 0.9 |  |  |  |  |
| Ilmn300K | 0.564 | 0.664 | 0.512 | 0.583 |
| Ilmn550K | 0.727 | 0.815 | 0.62 | 0.682 |
| Ilmn650K | 0.758 | 0.838 | 0.643 | 0.701 |

**Supplemental Table 1(f). Genetic Coverage of tagSNP Arrays on AffxSNPs that 50%>MAF≥35%**

|  | Affx HapMap SNP | | Affx NonHapMap SNP | |
| --- | --- | --- | --- | --- |
| r^2^_cutoff_ = 0.8 | Liver Study Caucasian | CEU | Liver Study Caucasian | CEU |
| Ilmn300K | 0.728 | 0.827 | 0.671 | 0.761 |
| Ilmn550K | 0.846 | 0.921 | 0.749 | 0.825 |
| Ilmn650K | 0.862 | 0.928 | 0.760 | 0.832 |
| r^2^_cutoff_ = 0.9 |  |  |  |  |
| Ilmn300K | 0.573 | 0.671 | 0.508 | 0.592 |
| Ilmn550K | 0.734 | 0.820 | 0.619 | 0.690 |
| Ilmn650K | 0.765 | 0.843 | 0.642 | 0.709 |
