# Supplementary material for: Calibrating the Performance of SNP Arrays for Whole-Genome Association Studies
Source: PLoS Genet. 2008 Jun 27;4(6):e1000109. doi: 10.1371/journal.pgen.1000109 (PMC2432039; doi:10.1371/journal.pgen.1000109)
Supplement: Table S2 — Power and Number of Discoveries (ND) of Whole Genome SNP Genotyping Products. (0.21 MB DOC) [file pgen.1000109.s007.doc]

**Supplemental Table 2. Power and Number of Discoveries (ND) of Whole Genome SNP Genotyping Products**

1. **Quantitative Trait ~ N (μ, σ^2^), μ = [-0.25, 0, 0.25] and σ = 1**

| **Affx500K** | Kruskal-Wallis Test | | | | Spearman Correlation Test | | | |
| --- | --- | --- | --- | --- | --- | --- | --- | --- |
|  | NonHapMap QTL | | HapMap QTL | | NonHapMap QTL | | HapMap QTL | |
| **α** | Power | NTD | Power | NTD | Power | NTD | Power | NTD |
| 10^-5^ | 0.016 | 0.032 | 0.016 | 0.031 | 0.036 | 0.077 | 0.036 | 0.075 |
| 10^-6^ | 0.004 | 0.008 | 0.004 | 0.008 | 0.012 | 0.024 | 0.012 | 0.024 |
| 10^-7^ | 0.001 | 0.002 | 0.001 | 0.002 | 0.004 | 0.007 | 0.004 | 0.007 |
|  |  | |  | |  | |  | |
| **Ilmn300K** |  | |  | |  | |  | |
| **α** | Power | NTD | Power | NTD | Power | NTD | Power | NTD |
| 10^-5^ | 0.019 | 0.030 | 0.025 | 0.042 | 0.039 | 0.068 | 0.050 | 0.092 |
| 10^-6^ | 0.005 | 0.008 | 0.007 | 0.011 | 0.013 | 0.021 | 0.018 | 0.029 |
| 10^-7^ | 0.001 | 0.002 | 0.002 | 0.003 | 0.004 | 0.009 | 0.005 | 0.006 |
|  |  | |  | |  | |  | |
| **Ilmn550K** |  | |  | |  | |  | |
| **α** | Power | NTD | Power | NTD | Power | NTD | Power | NTD |
| 10^-5^ | 0.022 | 0.046 | 0.029 | 0.058 | 0.045 | 0.103 | 0.058 | 0.130 |
| 10^-6^ | 0.006 | 0.012 | 0.008 | 0.016 | 0.015 | 0.032 | 0.021 | 0.041 |
| 10^-7^ | 0.002 | 0.003 | 0.002 | 0.004 | 0.005 | 0.010 | 0.007 | 0.012 |
|  |  | |  | |  | |  | |
| **Ilmn650K** |  | |  | |  | |  | |
| **α** | Power | NTD | Power | NTD | Power | NTD | Power | NTD |
| 10^-5^ | 0.023 | 0.052 | 0.031 | 0.068 | 0.048 | 0.119 | 0.060 | 0.148 |
| 10^-6^ | 0.007 | 0.014 | 0.009 | 0.019 | 0.015 | 0.037 | 0.022 | 0.048 |
| 10^-7^ | 0.002 | 0.003 | 0.003 | 0.005 | 0.005 | 0.011 | 0.007 | 0.015 |

1. **Quantitative Trait ~ N (μ, σ^2^), μ = [-0.5, 0, 0.5] and σ = 1**

| **Affx500K** | Kruskal-Wallis Test | | | | Spearman Correlation Test | | | |
| --- | --- | --- | --- | --- | --- | --- | --- | --- |
|  | NonHapMap QTL | | HapMap QTL | | NonHapMap QTL | | HapMap QTL | |
| **α** | Power | NTD | Power | NTD | Power | NTD | Power | NTD |
| 10^-5^ | 0.40 | 1.29 | 0.40 | 1.30 | 0.48 | 1.67 | 0.48 | 1.69 |
| 10^-6^ | 0.29 | 0.82 | 0.28 | 0.82 | 0.37 | 1.12 | 0.37 | 1.13 |
| 10^-7^ | 0.20 | 0.51 | 0.19 | 0.50 | 0.27 | 0.74 | 0.27 | 0.74 |
|  |  | |  | |  | |  | |
| **Ilmn300K** |  | |  | |  | |  | |
| **α** | Power | NTD | Power | NTD | Power | NTD | Power | NTD |
| 10^-5^ | 0.46 | 1.11 | 0.50 | 1.21 | 0.54 | 1.48 | 0.58 | 1.58 |
| 10^-6^ | 0.33 | 0.70 | 0.38 | 0.77 | 0.41 | 0.98 | 0.46 | 1.07 |
| 10^-7^ | 0.23 | 0.42 | 0.26 | 0.48 | 0.31 | 0.63 | 0.35 | 0.72 |
|  |  | |  | |  | |  | |
| **Ilmn550K** |  | |  | |  | |  | |
| **α** | Power | NTD | Power | NTD | Power | NTD | Power | NTD |
| 10^-5^ | 0.50 | 1.69 | 0.56 | 1.91 | 0.58 | 2.26 | 0.64 | 2.52 |
| 10^-6^ | 0.37 | 1.06 | 0.43 | 1.23 | 0.46 | 1.49 | 0.52 | 1.70 |
| 10^-7^ | 0.25 | 0.64 | 0.31 | 0.76 | 0.35 | 0.98 | 0.40 | 1.13 |
|  |  | |  | |  | |  | |
| **Ilmn650K** |  | |  | |  | |  | |
| **α** | Power | NTD | Power | NTD | Power | NTD | Power | NTD |
| 10^-5^ | 0.51 | 1.94 | 0.56 | 2.18 | 0.59 | 2.59 | 0.64 | 2.86 |
| 10^-6^ | 0.38 | 1.22 | 0.43 | 1.39 | 0.47 | 1.71 | 0.52 | 1.93 |
| 10^-7^ | 0.26 | 0.74 | 0.31 | 0.87 | 0.35 | 1.12 | 0.40 | 1.29 |

**C. Quantitative Trait ~ N (μ, σ^2^), μ = [-1.0, 0, 1.0] and σ = 1**

| **Affx500K** | Kruskal-Wallis Test | | | | Spearman Correlation Test | | | |
| --- | --- | --- | --- | --- | --- | --- | --- | --- |
|  | NonHapMap QTL | | HapMap QTL | | NonHapMap QTL | | HapMap QTL | |
| **α** | Power | NTD | Power | NTD | Power | NTD | Power | NTD |
| 10^-5^ | 0.69 | 3.32 | 0.70 | 3.30 | 0.71 | 3.62 | 0.72 | 3.58 |
| 10^-6^ | 0.65 | 2.91 | 0.66 | 2.92 | 0.68 | 3.16 | 0.68 | 3.15 |
| 10^-7^ | 0.62 | 2.62 | 0.63 | 2.62 | 0.64 | 2.83 | 0.65 | 2.83 |
|  |  | |  | |  | |  | |
| **Ilmn300K** |  | |  | |  | |  | |
| **α** | Power | NTD | Power | NTD | Power | NTD | Power | NTD |
| 10^-5^ | 0.81 | 3.87 | 0.88 | 4.61 | 0.83 | 4.37 | 0.89 | 5.13 |
| 10^-6^ | 0.78 | 3.20 | 0.85 | 3.91 | 0.80 | 3.62 | 0.86 | 4.36 |
| 10^-7^ | 0.74 | 2.72 | 0.82 | 3.37 | 0.77 | 3.08 | 0.84 | 3.77 |
|  |  | |  | |  | |  | |
| **Ilmn550K** |  | |  | |  | |  | |
| **α** | Power | NTD | Power | NTD | Power | NTD | Power | NTD |
| 10^-5^ | 0.86 | 5.68 | 0.93 | 6.63 | 0.88 | 6.41 | 0.94 | 7.36 |
| 10^-6^ | 0.83 | 4.70 | 0.90 | 5.62 | 0.85 | 5.31 | 0.92 | 6.25 |
| 10^-7^ | 0.79 | 3.97 | 0.87 | 4.85 | 0.82 | 4.50 | 0.89 | 5.41 |
|  |  | |  | |  | |  | |
| **Ilmn650K** |  | |  | |  | |  | |
| **α** | Power | NTD | Power | NTD | Power | NTD | Power | NTD |
| 10^-5^ | 0.87 | 6.30 | 0.93 | 7.39 | 0.89 | 7.09 | 0.94 | 8.19 |
| 10^-6^ | 0.84 | 5.23 | 0.90 | 6.29 | 0.86 | 5.90 | 0.92 | 6.98 |
| 10^-7^ | 0.80 | 4.44 | 0.88 | 5.43 | 0.83 | 5.02 | 0.90 | 6.05 |

1. **Binary Trait (Multiplicative Genetic Model, Relative Risk =3)**

|  | **Affx500K** | | | | **Ilmn300K** | | | |
| --- | --- | --- | --- | --- | --- | --- | --- | --- |
|  | NonHapMap QTL | | HapMap QTL | | NonHapMap QTL | | HapMap QTL | |
| **α** | Power | NTD | Power | NTD | Power | NTD | Power | NTD |
| 10^-5^ | 0.55 | 1.90 | 0.55 | 1.93 | 0.61 | 1.57 | 0.64 | 1.63 |
| 10^-6^ | 0.44 | 1.31 | 0.43 | 1.32 | 0.48 | 1.04 | 0.51 | 1.09 |
| 10^-7^ | 0.33 | 0.89 | 0.32 | 0.88 | 0.36 | 0.68 | 0.39 | 0.72 |
|  | **Ilmn550K** | | | | **Ilmn650K** | | | |
|  | NonHapMap QTL | | HapMap QTL | | NonHapMap QTL | | HapMap QTL | |
| **α** | Power | NTD | Power | NTD | Power | NTD | Power | NTD |
| 10^-5^ | 0.65 | 2.41 | 0.70 | 2.62 | 0.66 | 2.76 | 0.70 | 2.98 |
| 10^-6^ | 0.52 | 1.60 | 0.57 | 1.76 | 0.53 | 1.84 | 0.58 | 2.01 |
| 10^-7^ | 0.39 | 1.04 | 0.44 | 1.16 | 0.40 | 1.20 | 0.45 | 1.33 |

359 Caucasian subjects were used to investigate genotyping panels' statistical power. In table A, B and C, we assumed a quantitative trait follows normal distribution N(μ, σ^2^), where σ=1 and μ was different among genotypes. In Table A, μ_AA_=0.25, μ_Aa_= 0 and μ_aa_ = -0.25; in Table B, μ_Aa_ = 0.5, μ_Aa_= 0 and μ_aa_ = -0.5; in Table C, μ_Aa_ = 1, μ_Aa_= 0 and μ_aa_ =-1. In each simulation loop, we randomly selected a SNP from Affx500K and used its genotype to simulate trait value. Afterwards, we conducted Kruskal-Wallis and Spearman correlation tests to detect the phenotype-genotype association. The very SNP used in simulating phenotype was excluded from testing. In Table D, we generate case-ctrl status using a multiplicative model, where disease prevalence = 25% and relative risk = 3. 75 cases and 75 controls were used in simulation study (fisher's exact test was applied). We defined true discoveries as associations detected within 200kb of the causal SNP. 2 million simulation loops were run for each parameter setting. **Power** was the proportion that the QTL was detected by at least one nearby SNP on the microarray, and **NTD** (number of true discoveries) was the average true discoveries detected in each simulation. To study the over-fitting of Ilumina's tag SNP panels, we stratified the Affx500K SNPs into two strata (1) Affx HapMap SNPs and (2) Affx NonHapMap SNPs.
