# Supplementary material for: Calibrating the Performance of SNP Arrays for Whole-Genome Association Studies
Source: PLoS Genet. 2008 Jun 27;4(6):e1000109. doi: 10.1371/journal.pgen.1000109 (PMC2432039; doi:10.1371/journal.pgen.1000109)
Supplement: Table S3 — Number of False Discoveries in Mapping Quantitative Trait. (0.04 MB DOC) [file pgen.1000109.s008.doc]

**Supplementary Table 3. Number of False Discoveries in Mapping Quantitative Trait**

|  | **Affx500K** | | **Ilmn300K** | | **Ilmn550K** | | **Ilmn650K** | |
| --- | --- | --- | --- | --- | --- | --- | --- | --- |
| **SNP used in Analysis** | 286K | | 296K | | 514K | | 545K | |
| **α** | Kruskal | Spearmen | Kruskal | Spearmen | Kruskal | Spearmen | Kruskal | Spearmen |
| 10^-5^ | 1.56 | 2.76 | 1.97 | 3.42 | 2.61 | 5.01 | 2.84 | 5.14 |
| 10^-6^ | 0.14 | 0.34 | 0.16 | 0.36 | 0.20 | 0.54 | 0.21 | 0.56 |
| 10^-7^ | 0.01 | 0.03 | 0.02 | 0.03 | 0.02 | 0.06 | 0.01 | 0.06 |

359 Caucasian subjects were used to investigate genotyping panels' false discovery number. We assumed a quantitative trait following normal distribution N(μ, σ^2^), where σ^2^=1 and μ_Aa_ = 0.5, μ_Aa_= 0, μ_aa_ = -0.5. Simulation procedure was identical as in Table S2, and we presented the average number of false discoveries that were detected > 1Mb away from the causal SNP or on different chromosome from the causal SNP. We found that the number of false discovery (NFD) was similar for HapMap QTLs and NonHapMap QTLs. Therefore, we displayed the average NFD in the table.
