# Supplementary material for: Calibrating the Performance of SNP Arrays for Whole-Genome Association Studies
Source: PLoS Genet. 2008 Jun 27;4(6):e1000109. doi: 10.1371/journal.pgen.1000109 (PMC2432039; doi:10.1371/journal.pgen.1000109)
Supplement: Text S1 — Supplementary Materials. (0.03 MB DOC) [file pgen.1000109.s009.doc]

**Supplementary Methods**

RNA preparation and array hybridizations were performed at Rosetta Inpharmatics. The custom ink-jet microarrays used in this study were manufactured by Agilent Technologies (Palo Alto, CA) and consisted of 4,720 control probes and 39,280 non-control oligonucleotides extracted from mouse Unigene clusters and combined with RefSeq sequences and RIKEN full-length cDNA clones

Liver samples extracted from Caucasian individuals were homogenized and total RNA extracted using TRIzol reagent (Invitrogen, Carlsbad, California, United States) according to manufacturer's protocol. Three micrograms of total RNA was reverse transcribed and labeled with either Cy3 or Cy5 fluorochrome. Purified Cy3 or Cy5 complementary RNA was hybridized to at least two single microarrays with fluor- reversal for 24 h in a hybridization chamber, washed, and scanned using a laser confocal scanner. Arrays were quantified on the basis of spot intensity relative to background, adjusted for experimental variation between arrays using average intensity over multiple channels, and fitted to an error model to determine significance (type I error), as previously described.^1^ Gene expression is reported as the mean-log ratio relative to the pool derived from 192 liver samples selected for gender balance from the Vanderbilt and Pittsburgh samples as the RNA from the Merck samples had been amplified at an earlier date. The error model used to assess whether a given gene is significantly differentially expressed in a single sample relative to a pool comprised of a randomly selected subset of samples has been extensively described and tested in a number of publications^1-3^. Before performing association tests, we adjusted the expression traits for gender, age, and medical center using robust linear models.

***References***

1. He, Y. D. et al. Microarray standard data set and figures of merit for comparing data

processing methods and experiment designs. Bioinformatics 19, 956-65 (2003).

2. Hughes, T. R. et al. Functional discovery via a compendium of expression profiles. Cell 102, 109-26. (2000).

3. Roberts, C. J. et al. Signaling and circuitry of multiple MAPK pathways revealed by a matrix of global gene expression profiles. Science 287, 873-80 (2000).
